# Post–Caesarean Section Bilateral Cortical Blindness in a Primigravida With Systemic Lupus Erythematosus: A Case Report

**DOI:** 10.1155/crnm/2677412

**Published:** 2025-12-02

**Authors:** Athar Rasekh Jahromi, Reza Sahraei, Mehrdad Mahdavi, Fatemeh Rezaeian, Mohammad Fereidouni, Fatemeh Amiri

**Affiliations:** ^1^Department of Obstetrics and Gynecology, Jahrom University of Medical Sciences, Jahrom, Iran; ^2^Department of Anesthesiology, Jahrom University of Medical Sciences, Jahrom, Iran; ^3^Student Research Committee, Jahrom University of Medical Sciences, Jahrom, Iran; ^4^Department of Neurology, Jahrom University of Medical Sciences, Jahrom, Iran

**Keywords:** cortical blindness, posterior reversible encephalopathy syndrome, pregnancy, systemic lupus erythematosus

## Abstract

Systemic lupus erythematosus (SLE) increases the risk of morbidities during pregnancy, including stroke, thrombophilia, and antepartum bleeding. There are few case reports in English literature where bilateral cortical blindness has been described for a pregnant subject with prior SLE. In this report, a 32-year-old primigravida, a known case of SLE, was admitted with premature rupture of membranes at 34 weeks of gestation. Three hours after admission, due to vaginal bleeding, a healthy baby was delivered through emergency cesarean section under general anesthesia because of recent use of anticoagulants. In the recovery room, an elevated systolic blood pressure of 180 mmHg was noticed and controlled. After recovery, the patient complained of severe blurred vision. There were no abnormal findings on ophthalmologic examination but her brain MRI revealed bilateral occipital lesions. Bilateral cortical blindness is a rare incident during the pregnancy of an SLE patient without preeclampsia or eclampsia. Differential diagnoses, considering all the evidences, were posterior reversible encephalopathy syndrome (PRES) and ischemic stroke. Finally, we discuss the challenges of making a definitive final diagnosis, the importance of close follow-up for such cases, and control of underlying disease before pregnancy in such cases.

## 1. Introduction

Systemic lupus erythematosus (SLE) is a chronic autoimmune disease with an incidence ranging from 0.3 to 23.2 per 100,000 person-years, that primarily affects women of reproductive age [1, 2]. SLE is characterized by the production of autoantibodies against various cellular components, leading to widespread inflammation and potential damage to multiple organs, including the brain [3–6].

When pregnancy occurs in the context of SLE, it may not only be associated with an increased risk of lupus flares [7] but also with a higher likelihood of cesarean delivery, preterm labor, preeclampsia, and thrombophilia compared to the general obstetric population [8].

Among pregnant women, primigravidae are at a particularly elevated risk for lupus flare-ups compared to multigravid patients [9]. Furthermore, SLE itself can manifest with a wide spectrum of neuropsychiatric disorders, ranging from mood disturbances to cerebrovascular disease [10]. In rare cases, SLE can lead to neurological complications, such as bilateral cortical blindness (BCB) [11–15].

Posterior reversible encephalopathy syndrome (PRES) is a clinicoradiological entity characterized by a distinct constellation of neurological symptoms, including headache, visual disturbances, seizures, and altered mental status. The diagnosis is substantiated by specific neuroimaging findings, typically observed on magnetic resonance imaging (MRI). This syndrome frequently arises in the context of predisposing conditions such as hypertension, eclampsia, and autoimmune diseases including SLE, scleroderma, and rheumatoid arthritis and exposure to certain medications such as corticosteroids, tacrolimus, methotrexate, and azathioprine among many others [16].

Cortical blindness, though most frequently linked to preeclampsia and eclampsia in non-lupus pregnancies [17, 18], is a rare occurrence in nonpregnant SLE patients—especially in its bilateral presentation [19]. We present an unusual case combining these distinctive features: a primigravida with pre-existing SLE who developed BCB without evidence of preeclampsia. This report underscores the potential for severe and uncommon neurological manifestations in pregnant patients with SLE, emphasizing the need for heightened clinical vigilance.

## 2. Case Presentation

A 32-year-old primigravida woman, with SLE disease in her 34 weeks of gestation, was admitted to the emergency department of a southern hospital in Iran following a premature rupture of membrane from 3 h ago without history of trauma. She was a midwife by profession, and her statements were reliable.

She mentioned taking 40 mg enoxaparin subcutaneously and 80 mg of oral aspirin 4 h ago. These medications have been prescribed since her pregnancy. She also took daily sertraline, daily buspirone, oral prednisolone daily, oral azathioprine twice a day, oral tacrolimus twice a day, and oral hydroxychloroquine twice a day for her SLE. Prepregnancy rheumatological records were not available.

On admission, she was alert and her vital signs including blood pressure (BP) were normal and stable. Cervical dilation was 1 cm without effacement and minimal vaginal bleeding. A fetal heart rate of 142 bpm and a nonstress test without acceleration were recorded.

Before or during pregnancy, she had no history of seizures, no altered mental status, no visual disturbances, and no history of abnormally recurrent severe headaches although she mentioned arthralgia and arthritis. She also had not been visited by her rheumatologist for the past 6 months. Her daily sertraline and buspirone was started about 2 years ago. Examinations in the ward revealed no rash or ulcers anywhere, no evidence of vasculitis or arthritis, no cranial nerves neuropathy, and no fever.

The 24-h urine protein test at 27 weeks of gestation was 195 mg, but normal at 32 weeks. Amniotic fluid index was 25 cm (polyhydramnios) in ultrasonography at 33 weeks of gestational age. Previous autoantibody tests were positive for ANA, anti-ds-DNA, and anti-DNA IgG, but there was no previous laboratory record of antiphospholipid syndrome (APS). Her platelet count, prothrombin time, and partial thromboplastin time on admission were 163 × 10^3^/μL, 12.6 s and 38 s, respectively, with an international normalized ratio (INR) of 1.1. In hospital CBC was normal, serum creatinine was 0.9 mg/dL, BUN was 12 mg/dL, and her urine analysis showed trace blood with negative protein and 2-3 WBC.

A moderate vaginal bleeding episode occurred 3 h later, and an emergency C/S was planned. The patient received a perioperative stress dose of steroids and underwent surgery under general anesthesia due to recent use of anticoagulants. During the operation, there was minimal bleeding, and vital signs were normal. A neonate girl was born with a birth weight of 2500 g and a first-minute APGAR of 9/10.

After the surgery, due to the absence of spontaneous breathing, despite administration of atropine and neostigmine for the reversal of anesthesia, the patient remained intubated in the operating room. While intubated, the BP rose to 180/100, and a single dose of 5 mg of IV labetalol was administered, which brought the BP down to 130/80.

A Neurologist and Internist were consulted to determine whether there was a possibility of drug side effects, cerebrovascular events, or SLE-related complications. The consultation led to the ordering of necessary laboratory tests, imaging, and transfer to the intensive care unit (ICU). Initial arterial blood gas (ABG) test at ICU had a pH of 7.48 with a HCO_3_ of 20.3 mmol/L, PCO2 of 23.4, and PO2 of 110.

**Figure 1:**
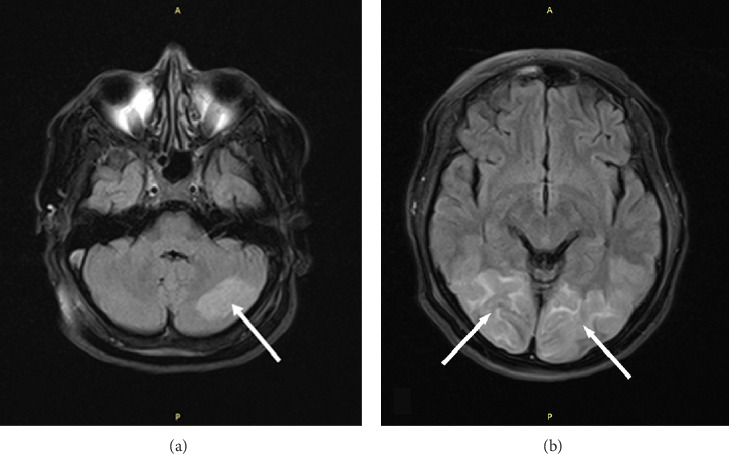
FLAIR sequence screening showed increased signal intensity in the left cerebellar hemisphere ((a) white arrow) and posterior occipital regions ((b) white arrows).

**Figure 2:**
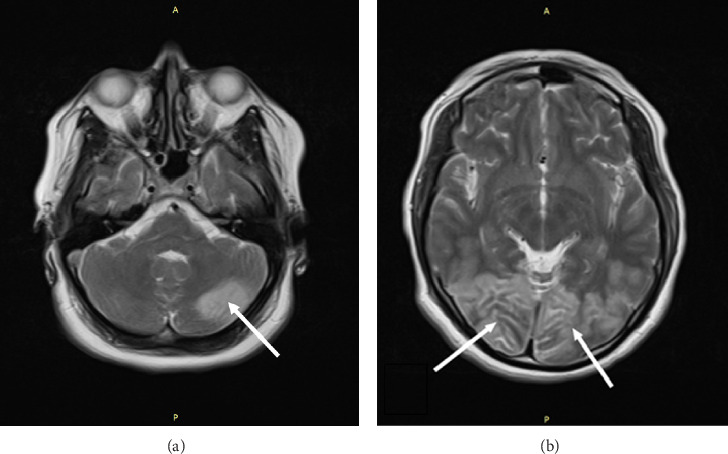
A T2 sequence screening showed hypersignal intensity in the cortex and subcortical areas of the bilateral occipital lobes ((b) white arrows) along with the posterior side of the left cerebellum ((a) white arrow).

**Figure 3:**
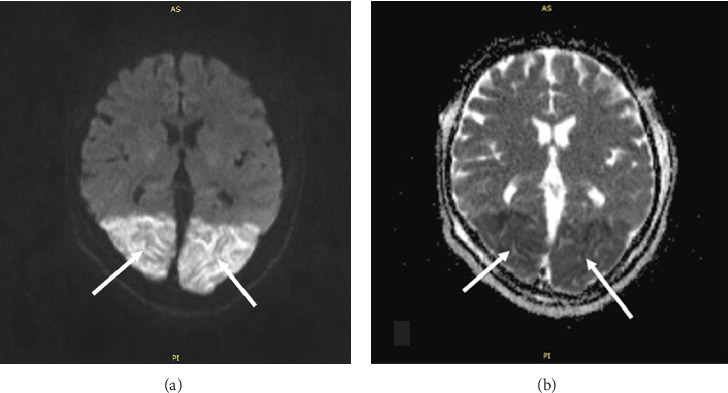
DWI (a) ADC and (b) sequences indicating ischemic infarction by diffusion restriction (white arrows).

Seven hours later, the patient was extubated and admitted to the ICU. In the ICU, after reawakening, the patient complained of severe blurred vision and had only perception of light. An ophthalmologist noted normal pupillary reflex and size and did not notice any abnormal findings on fundoscopy. After consulting a neurologist, she noted that there were no focal neurological deficits, but her MRI indicated bilateral occipital infarctions that extend to the posterior cerebellar hemisphere, explaining her blurred vision (Figures 1, 2, and 3).

No abnormalities were observed in the results of brain and neck magnetic resonance venography (MRV), magnetic resonance angiography (MRA), and color Doppler sonography (CDS) of the lower extremities, carotid arteries, and vertebral arteries.

There were no other high BP episodes during the ICU admission. The patient's medications included subcutaneous enoxaparin 40 mg once daily with coagulation profile monitoring, IV hydrocortisone 100 mg divided into three doses, and hydroxychloroquine 200 mg tablets each night. D-Dimer was negative, liver function tests (LFT) were normal, and the only abnormal laboratory test was U/A with protein 1+, blood 2+, and RBC = 28. A day later, she was started on rituximab and prednisolone pulse and maintenance dose because of possibility of active neuropsychiatric lupus at the time.

After 3 weeks, the patient reports detecting movements in front of her at a distance of 1 m. In addition, the patient reports that she can distinguish some colors and the presence of light. However, her vision has not improved significantly. Three months later, as per verbal follow-ups, the patient reads the time or text messages on her phone. However, she experiences peripheral blurriness in her visual field and perceives a narrower field of vision.

## 3. Discussion

This case report presents a 32-year-old woman with known SLE who became pregnant for the first time. An emergency C/S was necessitated at 34 weeks of gestation due to a rupture of membrane followed by vaginal bleeding. The patient experienced an episode of high BP in the operating room and was not extubated until a few hours after the surgery due to the lack of spontaneous breathing. After fully regaining consciousness from general anesthesia, the patient complained of severe visual disturbance and blurriness. Our top differential diagnoses for cortical blindness in this patient include ischemic cerebrovascular accident (CVA) and PRES. We have also considered severe preeclampsia and reversible cerebral vasoconstriction syndrome (RCVS) as other possible diagnoses. Based on clinical history and imaging of the patient, especially an absence of thunderclap headaches and a normal MRA, we found RCVS an unlikely explanation for the patient's symptoms [20].

As for the severe preeclampsia differential, although there was a new-onset visual disturbance but since there was only a single episode of high BP and a 24-h urine protein of less than 300 mg without any pulmonary edema, and considering that the patient's LFT, platelet count, and serum creatinine were also normal, we were able to reasonably rule out the said differential [21].

In a study conducted by Clowse et al., the prevalence of hypertension in SLE pregnancies compared to nonlupus pregnancies was 3.9% versus 0.7%, respectively. The ratio for thrombophilia was 4%, compared to 0.04%. Furthermore, the incidence of preeclampsia and caesarean section was significantly higher in pregnant mothers with SLE than those without SLE. Also, stroke and antepartum bleeding incidence in SLE pregnancies was 0.32% and 2%, respectively. In contrast, these complications in non-lupus pregnancies were 0.03% and 0.4%, respectively [8].

Neuropsychiatric involvements in SLE can manifest both in the central nervous system (CNS) and the peripheral nervous system (PNS), presenting with a wide spectrum of manifestations, including cerebrovascular disease, cognitive and psychiatric disorders, seizure disorders, and Myasthenia gravis [10].

Limited studies have been conducted on cortical blindness prevalence in pregnancies without SLE. Cortical blindness in a nonlupus pregnancy is typically associated with preeclampsia and eclampsia, with reported prevalence rates ranging widely from 0.15% to 15% [17, 18]. In a SLE patient, the most common ocular manifestation is keratoconjunctivitis sicca [22]. The exact prevalence of cortical blindness in SLE is not precisely determined. However, numerous reports are indicating transient or reversible cortical blindness in SLE patients due to PRES [19].

One study estimated that 0.69% percent of SLE patients had PRES [23]. High BP, immunomodulatory drugs such as tacrolimus, autoimmune diseases such as SLE and APS, preeclampsia and eclampsia, and steroids use can all contribute to the PRES phenomenon [13].

Important imaging features of PRES include diffuse abnormal changes, mainly in the posterior regions of the brain's white matter, that are manifested as hypodense on CT, hyperintense on T2-weighted MRI, and alterations in diffusion-weighted imaging (DWI) and apparent diffusion coefficient (ADC) maps [24].

Clinical symptoms of PRES include altered level of consciousness, seizures, headache, and visual disturbances, including cortical blindness [25].

The pathogenesis of PRES is not fully understood, but it appears that a sudden increase in BP disrupts cerebral blood vessels autoregulation, particularly in the occipital lobes where sympathetic innervation is less, leading to vasogenic edema in that area. In addition, another hypothesis suggests that damage and dysfunction of endothelial cells are involved in pathogenesis of PRES [26]. However, the pathology is not always limited to the occipital or posterior regions of the brain, and the involvement of other areas without posterior brain involvement are also possible [27]. Contrary to the name PRES, this syndrome is not always reversible and can be associated with permanent neurological deficits that sometimes lead to infarction in the mentioned areas [28, 29].

In our patient's brain MRI images, we observed increased signal intensity on T2 and FLAIR sequences in the posterior occipital regions as well as the left cerebellar hemisphere (Figures 1 and 2). Additionally, increased signal intensity on DWI and decreased signal intensity on ADC MAPS were observed in the same areas (Figure 3). The simultaneous presence of these signs on MRI suggests acute ischemia and infarction. On the other hand, in the presented patient, the presence of an acute episode of hypertension along with the use of immunosuppressive drugs, corticosteroids, and confirmed SLE as an underlying condition are factors that could predispose the individual to PRES. In addition, the patient clinical symptoms, including confusion and cortical visual impairments are consistent with the common clinical features of PRES. However, the patient did not experience a seizure episode, which could be due to the prophylactic administration of magnesium sulfate or masking with general anesthesia. Another discrepancy between the patient's clinical condition and what is typical of PRES is the lack of full visual recovery after 2 and a half months from the incident. However, as mentioned above, PRES is not always reversible.

As depicted in the MRI images of this patient, involvement of the left cerebellar hemisphere is also evident. Cerebellar involvement in PRES is less common, but it is noteworthy that in a study published by Fugate et al., cerebellar involvement in PRES was significantly more pronounced in patients with a history of autoimmune diseases [30].

Based on the radiographic images of this patient, the occurrence of acute infarction in the posterior regions is evident, as indicated by an increase in signal on DWI and a decrease in ADC. for our team, it remains an unresolved question whether this infarction was initiated by an ischemic stroke due to vasculitis or thromboembolism, or if it was due to PRES which subsequently progressed to infarction but based on all the aforementioned evidences, the most plausible final diagnosis would be BCB secondary to PRES.

Our case illustrated a unique complication in the setting of SLE and pregnancy, permanent cortical blindness resulting from PRES-related infarction. Through this case, we aimed to contribute to the existing knowledge about rare neurological complication of pregnancies in the setting of SLE and also underline that the effective management of such multifaceted conditions is reliant on a coordinated, multidisciplinary approach. Ultimately, in the event of a pregnancy in the setting of SLE, considering the various risks that SLE poses during pregnancy, the patients should be closely monitored and followed up by an obstetrician specializing in SLE pregnancies and a rheumatologist. Ideally, these patients should avoid pregnancy unless their underlying disease is controlled.

## Data Availability

The datasets generated and/or analyzed during the current study are not publicly available due to concerns regarding individual privacy but are available from the corresponding author on reasonable request.

## Nomenclature

SLE: Systemic lupus erythematosus
PRES: Posterior reversible encephalopathy syndrome
CVA: Cerebrovascular accident
MRI: Magnetic resonance imaging
APS: Antiphospholipid syndrome
DWI: Diffusion weighted imaging
ADC: Apparent diffusion coefficient
FLAIR: Fluid-attenuated inversion recovery
CNS: Central nervous system
PNS: Peripheral nervous system
CDS: Color Doppler sonography
MRA: Magnetic resonance angiography
MRV: Magnetic resonance venography
ICU: Intensive care unit
BCB: Bilateral cortical blindness
C/S: Cesarean section
CT: Computed tomography
WBC: White blood cells
INR: International normalized ratio
CBC: Complete blood count
ABG: Arterial blood gas test
RCVS: Reversible cerebral vasoconstriction syndrome
LFT: Liver function tests
